# Supplementary material for: Comparison of regulatory approval system for medicines in emergency among Japan, the United States, the United Kingdom, Europe, and China
Source: PLoS One. 2024 Sep 16;19(9):e0309992. doi: 10.1371/journal.pone.0309992 (PMC11404807; doi:10.1371/journal.pone.0309992)
Supplement: Attachment — Submitted filename: Response_to_Reviwers_240821.docx [file pone.0309992.s002.docx]

Response to Reviewers

(Manuscript ID: PONE-D-24-08507])

Dear editor and reviewers,

Thank you for the time you invested in reviewing our manuscript. We are grateful for your thoughtful comments and suggestions to the manuscript, which have enriched the manuscript and produced a better and more balanced account of the research. Please find detailed responses to your comments below.

I. Response to Academic Editor Comments

1. Please ensure that your manuscript meets PLOS ONE's style requirements at

<https://journals.plos.org/plosone/s/file?id=wjVg/PLOSOne_formatting_sample_main_body.pdf>　and

<https://journals.plos.org/plosone/s/file?id=ba62/PLOSOne_formatting_sample_title_authors_affiliations.pdf>

Our response:

Thank you for your comment. We have re-examined the style of PLOS ONE and have aligned our manuscript formatting accordingly.

2. We note that the grant information you provided in the ‘Funding Information’ and ‘Financial Disclosure’ sections do not match. When you resubmit, please ensure that you provide the correct grant numbers for the awards you received for your study in the ‘Funding Information’ section.

3. Thank you for stating the following financial disclosure:

"This study was partially funded by a grant from Research on Regulatory Science of Pharmaceuticals and Medical Devices, Health, Labour and Welfare Policy Research (21KC2006)"

4. Thank you for stating in your Funding Statement:

 "This study was partially funded by a grant from Research on Regulatory Science of Pharmaceuticals and Medical Devices, Health, Labour and Welfare Policy Research (21KC2006)"

Please provide an amended statement that declares *all* the funding or sources of support (whether external or internal to your organization) received during this study, as detailed online in our guide for authors at http://journals.plos.org/plosone/s/submit-now.  Please also include the statement “There was no additional external funding received for this study.” in your updated Funding Statement.

Please include your amended Funding Statement within your cover letter. We will change the online submission form on your behalf.

Our response:

Thank you for your pointing this out. This study was not supported by any grant, including "21KC2006". I have corrected this error and apologize for the confusion.

Funding, Line 32-33

[Before revision]

**Funding:** This study was partially funded by a grant from Research on Regulatory Science of Pharmaceuticals and Medical Devices, Health, Labour and Welfare Policy Research (21KC2006).

[After revision]

**Funding:** No funding was received for this study.

5. Thank you for stating the following in the Competing Interests section:

"M.K is the employee of Astellas Pharma Inc."

Please confirm that this does not alter your adherence to all PLOS ONE policies on sharing data and materials, by including the following statement: ""This does not alter our adherence to  PLOS ONE policies on sharing data and materials.” (as detailed online in our guide for authors **Conflicts of Interest:** MK is an employee of Astellas Pharma Inc. Other authors have no conflict of interest to declare.).  If there are restrictions on sharing of data and/or materials, please state these. Please note that we cannot proceed with consideration of your article until this information has been declared.

Please include your updated Competing Interests statement in your cover letter; we will change the online submission form on your behalf.

Our response:

Thank you for pointing out. There are no restrictions on the sharing of data and/or materials which adheres to PLOS ONE policies on the sharing data and materials. We will include the following statement: "This does not alter our adherence to  PLOS ONE policies on the sharing of data and materials.” MK is a PhD candidate of Department of Regulatory Science, Graduate School of Pharmaceutical Science, Meiji Pharmaceutical University.

Conflicts of Interest , Line 28-29

[Before revision]

Conflicts of Interest: MK is an employee of Astellas Pharma Inc. Other authors have no conflict of interest to declare.

[After revision]

Conflicts of Interest: M.K. is an employee of Astellas Pharma Inc. Other authors have no conflicts of interest to declare. This does not alter our adherence to PLOS ONE policies on the sharing of data and materials.

6. PLOS requires an ORCID iD for the corresponding author in Editorial Manager on papers submitted after December 6th, 2016. Please ensure that you have an ORCID iD and that it is validated in Editorial Manager. To do this, go to ‘Update my Information’ (in the upper left-hand corner of the main menu), and click on the Fetch/Validate link next to the ORCID field. This will take you to the ORCID site and allow you to create a new iD or authenticate a pre-existing iD in Editorial Manager. Please see the following video for instructions on linking an ORCID iD to your Editorial Manager account: <https://www.youtube.com/watch?v=_xcclfuvtxQ>

Our response:

Thank you for your instruction. Please refer to the ORCID iDs for the corresponding authors of this manuscript:

Hideki Maeda; 0000000201173961

Masahide Kawano; 0009000532896280

Corresponding author, Line 18-19

[Before revision]

Hideki Maeda (maeda@my-pharm.ac.jp) ORCID ID: 0000000201173961

[After revision]

Hideki Maeda (maeda@my-pharm.ac.jp) ORCID ID: 0000000201173961

Masahide Kawano (d226752@std.my-pharm.ac.jp) ORCID ID: 0009000532896280

7. Please upload a new copy of Figure xxxx as the detail is not clear. Please follow the link for more information: "<https://blogs.plos.org/plos/2019/06/looking-good-tips-for-creating-your-plos-figures-graphics/>" "<https://blogs.plos.org/plos/2019/06/looking-good-tips-for-creating-your-plos-figures-graphics/>"

Our response:

We appreciate your comment and apologize for the poor resolution of the figure as you indicated. Please find attached the corrected Figure and Table.

II. Response to Reviewer #1

The article "Comparison of regulatory approval system for medicines in emergency among Japan, the United States, the United Kingdom, Europe, and China" addresses an important and timely issue with respect to the variability in emergency drug regulatory approval process for various countries, focusing on pharmaceuticals related to COVID-19.

Unfortunately, in its current form I cannot recommend this article for publication. There are several significant concerns regarding elements of study design and statistical evaluation.

Details can be found in the respective sections to follow:

Introduction:

The authors provide a strong motivation for the work, highlighting the heterogeneity in the drug review processes across countries.

1. - More detail could be provided around the specific variances across each of the countries processes that will be discussed.

Our response:

Thank you for your valuable feedback. As you indicated, we have added details of the EU, UK, and CN systems to illustrate the differences regarding the process in each country. Regarding each section, we have prepared a response to each of the points you raised. Kindly refer to the responses below.

Introduction, Line 80-84

[Before revision]

… In Europe (EU) [6], the United Kingdom (UK) [7], and China (CH) [8], early approval and permission systems have been established as frameworks for using unapproved drugs during emergencies.

[After revision]

…In Europe (EU) [6], the United Kingdom (UK) [7], and China (CN) [8], early approval and permission systems have been established as frameworks for using unapproved drugs during emergencies. In the EU, conditional marketing authorization is valid for one year and renewable annually. In the UK temporary use authorization is valid for one year and can be transferred to a normal authorization. CN has conditional market approval and emergency use of vaccines.

2. There is also an overall lack of related work exploring variance between countries in related topics such as COVID-19 vaccine approval.

e.g.

Lythgoe, Mark P., and Paul Middleton. "Comparison of COVID-19 vaccine approvals at the US Food and Drug administration, European Medicines agency, and health Canada." JAMA Network Open 4.6 (2021): e2114531-e2114531.

Our response:

We agree with your insight and have therefore added six new references, including the paper you recommended (21-26). Additionally, we have sequentially numbered the references that appear after the original number 20.

References, Lines 403-413

[Before revision]

…

20. Enya K, Lim Y, Sengoku S, Kodama K. Increasing orphan drug loss in Japan: Trends and R&D strategy for rare diseases. Drug Discov Today. 2023 Aug 28;28(10):103755.

[After revision]

20. Enya K, Lim Y, Sengoku S, Kodama K. Increasing orphan drug loss in Japan: Trends and R&D strategy for rare diseases. Drug Discov Today. 2023 Aug 28;28(10):103755.
21. Lythgoe, Mark P., and Paul Middleton. "Comparison of COVID-19 vaccine approvals at the US Food and Drug administration, European Medicines agency, and health Canada." JAMA Network Open 4.6 (2021): e2114531-e2114531..
22. Alessandra Donati. "The Conditional Marketing Authorisation of Covid-19 Vaccines: A Critical Assessment under EU Law." European Journal of Health Law. 2022 Mar 04; doi: 10.1163/15718093-bja10065
23. Xingyue Zhu, and Yang Chen. "Bridging the new drug access gap between China and the United States and its related policies." Front Pharmacol. 2023; 14: 1296737. Published online 2024 Jan 8. doi: 10.3389/fphar.2023.1296737
24. Wu Yingxiong. "A review of China's compensation program for adverse reactions following immunization." Front Public Health. 2023 Aug 7:11:1211976. doi: 10.3389/fpubh.2023.1211976.
25. Kyle Thomson, Herschel Nachlis "Emergency Use Authorizations for Medical Products During the COVID-19 Pandemic." JAMA. 2020;324(13):1282-1283. doi:10.1001/jama.2020.16253
26. P O’Malley, J Rainford, A Thompson " Transparency during public health emergencies: from rhetoric to reality" 2009 Aug; 87(8): 614–618. doi: 10.2471/BLT.08.056689

3. Materials and Methods:

Data collection

- This section needs expansion. The "proprietary dataset" is not sufficiently detailed. While citations for the data sources are valuable, the authors note that data collection was "primarily conducted" through official webpages. How was the remainder of data collected?

- More detail is needed regarding data extraction. Where in each of the webpages were data elements extracted?

- From the links provided, was any criterion or processing applied to identified data elements to create the final dataset.

- The use of a single reviewer is concerning. Given manual data extraction, dual review should have been performed to confirm quality of data elements.

Our response:

Thank you for your important comments. We have supplemented and expanded the details of data collection as shown below. The data used were obtained from the official website and ClinicalTrials.gov. Additionally, the extracted data were double-checked by EK and MK to ensure accuracy, and the final decision was made by HM.

Materials and methods, 1. Data Collection, Line 104-133

[Before revision]

.…The specific investigation parameters included approval or usage permission status, approval or usage permission dates, JP approval application dates, expiration dates of approval or usage permission, review periods, the duration from EUA issuance to approval or usage permission date in each country, and the developmental stage. Data collection was primarily conducted through the official websites of regulatory authorities in each country [11–15].	Each website was reviewed, and the data were manually extracted and verified by a researcher.

[After revision]

.…The specific investigation parameters included approval or usage permission status, approval or usage permission dates, JP approval application dates, expiration dates of approval or usage permission, review periods, the duration from EUA issuance to approval or usage permission date in each country, and the developmental stage. Data collection was conducted through the official websites of the regulatory authorities in each country [11–15] and ClinicalTrials.gov. Each website was reviewed, and the data were manually extracted and verified by a researcher. Data on the status of approval/use authorization, date of approval/use authorization, date of JP approval, date of withdrawal of approval/use authorization, review period, and period from the date of EUA issuance to the date of approval/use authorization in each country were collected from the regulatory agency web sites in each country as shown below. We also collected data from ClinicalTrials.gov on the pre-market phase of the drug, the most recent data available as of July 31, 2023. Duplicates were eliminated in the collected data, formatted uniformly, and compiled into a database.

In the United States (US): Information was collected from Drugs@FDA, the Emergency Use Authorization (EUA) page of the FDA web site, and FDA press releases.

(<https://www.fda.gov/emergency-preparedness-and-response/mcm-legal-regulatory-and-policy-framework/emergency-use-authorization>)

In Japan (JP), information was collected from the Pharmaceuticals and Medical Drugs Directory (PMDA), the Ministry of Health, Labour and Welfare (MHLW) website, and MHLW press releases.

(<https://www.pmda.go.jp/PmdaSearch/iyakuSearch>, <https://www.mhlw.go.jp/index.html>)

Europe (EU): Information was collected from the Conditional Marketing Authorization (CMA) page of the EMA web site and EMA press release information.

(<https://www.ema.europa.eu/en/glossary-terms/conditional-marketing-authorisation>)

In United Kingdom (UK): Information was collected from MHRA Products Substance Index, MHRA web site page on Temporary Use authorization, and MHRA press release information.

(<https://www.gov.uk/government/publications/freedom-of-information-responses-from-the-mhra-week-commencing-14-march-2022/freedom-of-information-request-on-the-different-legal-classifications-for-vaccines-foi-21415>)

CHINA (CN): Information was collected from the official NMPA website, CENTER FOR DRUG EVALUATION, NMPA.

(<https://english.nmpa.gov.cn/index.html>, <https://english.nmpa.gov.cn/2019-07/19/c_389169.htm>)

Materials and Methods, 2. Content of investigation, Line 153-155

[Before revision]

This study was conducted in accordance with the Strengthening the Reporting of Observational Studies in Epidemiology (STROBE) reporting guidelines [16] for cross-sectional studies.

[After revision]

This study was conducted in accordance with the Strengthening the Reporting of Observational Studies in Epidemiology (STROBE) reporting guidelines [16] for cross-sectional studies. Kazuki Edo (KE) and Masahide Kawano (MK) independently conducted the investigation parameters. Any questions or issues were discussed between the two authors and were adjusted when necessary. Any disagreement was resolved by a third reviewer [Hideki Maeda (HM)].

4. - The hypothesis here does not seem to match the objectives outlined in the title/introduction. The article is positioned as a comparison of the approval of medications between multiple counties, however (A) clarifies this is restricted to only the subset of drugs approved by the United States EUA (and then comparing their approval in other countries). A full comparison between approval mechanisms would include the potential for drugs approved in other countries and not through the EUA. This is mentioned in 1 sentence for limitations, but this is not sufficient. Revision to the framing of the manuscript is needed.

--- This is also somewhat at odds with later analysis for timeline for EUA, in which some countries had negative timelines, suggesting their use of drug before FDA approval. The bi-directional framing of the analysis is needed.

Our response:

Thank you for your valuable comments. This study utilized extensive data from the U.S. EUA, which played an important role during the COVID-19 pandemic. We also used the U.S. EUA as our reference point to ensure consistent comparisons across countries. Due to the lack of explanation, we have added more details and corrected the information as follows:

Introduction, Lines 90-98

[Before revision]

…Therefore, this study aims to compare the emergency approval or usage systems for unapproved pharmaceuticals in JP, the US, the EU, the UK, and CH, focusing on investigating the approval and usage permission systems during emergencies such as pandemics, and aims to identify the commonalities and differences in each system. European countries under the European Union were considered as one country for the sake of this study. The study also explores how pharmaceuticals granted usage permission under the US EUA were approved or permitted in other countries (JP, EU, UK, and CH) and conducts a comparative analysis of the emergency approval or usage permission systems in each country. Finally, the study examines whether the differences in the systems for unapproved pharmaceuticals during emergencies in different countries affected drug access.

[After revision]

… Therefore, this study aimed to compare the emergency approval or usage systems for unapproved pharmaceuticals in JP, the US, the EU, the UK, and CN with reference to the U.S., focusing on investigating the approval and usage permission systems during emergencies such as pandemics, and aimed to identify the commonalities and differences in each system. European countries under the European Union were considered as one country for the purposes of this study. This study also explored how pharmaceuticals granted usage permission under the US EUA were approved or permitted in other countries (JP, EU, UK, and CN) and conducted a comparative analysis of the emergency approval or usage permission systems in each country. Finally, the study examined whether the differences in the systems for unapproved pharmaceuticals during emergencies in different countries affected drug access.

5. - The study does not appear to follow the complete STROBE guidelines. Please attach the checklist document with associated page numbers

Our response:

Thank you for your instruction. Please find attached the STROBE check list document.

6. It is unclear what had their "EUA permission continued" means, is this only those drugs continued with the July 2023 timeframe? Or continued at any point during this period after EUA. The timeframe is ambiguously defined, more information is needed.

Our response:

Thank you very much for sharing your important comments. We define "continuation of use permit by EUA" as the EUA permit continues to be issued as of July 31, 2023 and has not changed to full approval. We have added an explanation as follows:

Results, Lines 173-178

[Before revision]

… During the COVID-19 pandemic period (December 1, 2019, to July 31, 2023), 23 pharmaceuticals were granted usage permission under the US EUA. After re-evaluation by the FDA, five drugs (21.7%) received full approval, 13 drugs (56.5%) had their EUA usage permission continued, and five drugs (21.7%) had their EUA revoked or discontinued (Fig.1)...

[After revision]

… During the COVID-19 pandemic period (December 1, 2019, to July 31, 2023), 23 pharmaceuticals were granted usage permission under the US EUA. After re-evaluation by the FDA, five drugs (21.7%) received full approval, 13 drugs (56.5%) had their EUA usage permission continued, and five drugs (21.7%) had their EUA revoked or discontinued (Fig.1). "EUA usage permission continued" is defined as a drug in the US as of July 31, 2023, that has not yet been granted an EUA and has not moved to full approval. This definition includes all drugs that have been granted an EUA and remain in EUA status without having reached full approval by the specified date.

7. Analysis 1 (percentage of drugs approved by country - i.e. Table 1) appears flawed.

- First, drugs that are not approved in other countries, are listed as "withdrawal", however is it not possible these drugs were not even considered for emergency approval? The breakdown of only approved/withdraw seems problematic. Please expand/explain.

Our response:

Thank you for your attention to this important point. As you pointed out, the item "withdrawal" in Table 1 includes drugs that have not yet been developed, and we have revised the item "withdrawal" in Table 1 to "withdrawal or undeveloped" and modified Table 1 as shown in the attached file.

8. - The United States is included in the analysis. All other countries must consider equal to, or fewer total drugs as the superset of drugs is the 23 total drugs that obtained FDA approval. As we are considering non-approved drugs to be withdrawn it seems odd to include the United States in which all 23 drugs were approved with some withdrawn.

- This issue appears again in Table 2, which undeveloped drugs cannot exist for the US, yet are considered in the table.

Our response:

Thank you for your important feedback. This study included all medicines for which an EUA was issued in the US during the period covered (December 2019 - July 2023), and we investigated the status of these medicines in other countries. Therefore, we also considered medicinal products for which an EUA was once granted and subsequently withdrawn due to a subsequent review. In the US, all 23 medicinal products for which an EUA was issued were analyzed, some of which moved to full approval, while others continued as EUAs or were withdrawn or discontinued. This makes it possible to compare directly with the urgent approval systems of other countries on the basis of the US. We have made the following corrections, as explained below, due to the lack of explanation.

Results, Lines 185–189

[Before revision]

As of the end of July 2023, an investigation into the approval/usage permission status of pharmaceuticals granted usage permission under the EUA in other countries revealed that JP had obtained approval or permission for 14 drugs (60.9%), the EU for 14 drugs (60.9%), the UK for 12 drugs (52.2%), and CH for 3 drugs (13.0%). In comparison with each country, the US had a significantly highest number of usage permissions granted (P=0.0002, χ2 test) (Table 1).…

[After revision]

As of the end of July 2023, an investigation into the approval/usage permission status of medicinal products that had been previously licensed for use under the EUA in the period under study as of the end of July 2023 revealed that JP had obtained approval or permission for 14 drugs (60.9%), the EU for 14 drugs (60.9%), the UK for 12 drugs (52.2%), and CN for 3 drugs (13.0%). In comparison with each country, the US had a significantly highest number of usage permissions granted (P=0.0002, χ2 test) (Table 1).…

9. - Moreover Chi-squared is not an appropriate test for this data. The number of 0-cells is high. Fishers exact should have been performed.

Our response:

Thank you for your valuable suggestion. We have changed the statistical analysis method and performed Fishers exact test. The following corrections have been made:

Statistical methods, Lines 158-162

[Before revision]

Statistical analyses were performed using Microsoft Excel. T-tests were performed for the review periods in each country and the duration from the issuance date of the EUA to the approval date. For the developmental stages of each pharmaceutical in each country, χ2 tests were conducted. Statistical significance was set at 5% (two-tailed).

[After revision]

Microsoft® Excel and R-4.4.1 were used for statistical analysis. The Mann-Whitney U test was used for the review period and the period from the date of EUA issuance to the date of approval in each country, and the Fishers exact test was used for the development stage of each drug in each country. The statistical significance level was set at 5% (two-tailed). Bonferroni correction was applied to the Mann-Whitney U test to account for multiplicity.

Results, Lines 185–193

[Before revision]

As of the end of July 2023, an investigation into the approval/usage permission status of pharmaceuticals granted usage permission under the EUA in other countries revealed that JP had obtained approval or permission for 14 drugs (60.9%), the EU for 14 drugs (60.9%), the UK for 12 drugs (52.2%), and CH for 3 drugs (13.0%). In comparison with each country, the US had a significantly highest number of usage permissions granted (P=0.0002, χ2 test) (Table 1). Additionally, an investigation into the developmental status of pharmaceuticals granted usage permission under the EUA as of the end of July 2023 in each country showed that the EU had three drugs (8.7%), the UK had five drugs (21.7%), JP had six drugs (26.1%), and CH had 16 drugs (69.6%) that were yet to be developed. The proportion of undeveloped pharmaceuticals in CH was significantly larger compared to other countries (P<0.0001) (Table 2).

[After revision]

As of the end of July 2023, an investigation into the approval/usage permission status of pharmaceuticals granted usage permission under the EUA in other countries revealed that JP had obtained approval or permission for 14 drugs (60.9%), the EU for 14 drugs (60.9%), the UK for 12 drugs (52.2%), and CN for 3 drugs (13.0%). In comparison with each country, the US had a significantly highest number of usage permissions granted (P＝0.0001, Fishers exact test) (Table 1). Additionally, an investigation into the developmental status of pharmaceuticals granted usage permission under the EUA as of the end of July 2023 in each country showed that the EU had three drugs (8.7%), the UK had five drugs (21.7%), JP had six drugs (26.1%), and CN had 16 drugs (69.6%) that were yet to be developed. The proportion of undeveloped pharmaceuticals in CN was significantly larger compared to other countries (P<0.0001, Fishers exact test) (Table 2).

10. - Use of t-tests is somewhat odd. The authors report median/IRQ indicating awareness of a non-normal distribution (heavy tailed). As such, I would expect to see non-parametric Mann-U or similar tests used.

Our response:

Thank you very much for your valuable insight. We performed a non-parametric Mann-Whitney U test. Additionally, Bonferroni correction was performed in the Mann-Whitney U test to account for multiplicity. The following corrections have been made accordingly:

Statistical methods, Lines 158-162

[Before revision]

Statistical analyses were performed using Microsoft Excel. T-tests were performed for the review periods in each country and the duration from the issuance date of the EUA to the approval date. For the developmental stages of each pharmaceutical in each country, χ2 tests were conducted. Statistical significance was set at 5% (two-tailed).

[After revision]

Microsoft® Excel and R-4.4.1 were used for statistical analysis. The Mann-Whitney U test was used for the review period and the period from the date of EUA issuance to the date of approval in each country, and the Fishers exact test was used for the development stage of each drug in each country. The statistical significance level was set at 5% (two-tailed). Bonferroni correction was applied to the Mann-Whitney U test to account for multiplicity.

Result, Lines 205-208

[Before revision]

… The shortest review period was observed in the UK, where the mean was 50.5 days earlier (Fig.2), and a statistically significant difference in the review period was found between the US and the UK (P=0.02).

[After revision]

… The shortest review period was observed in the UK, where the mean was 50.5 days earlier (Fig.2). There was a statistically significant difference in the review period between US-UK (P=0.0037), JP-UK (P=0.0391). No statistically significant difference in examination duration was found between US-JP (P=0.9553), US-EU (P=0.4022), JP-EU (P=4.0866), and EU-UK (P=0.1509).

11. - Figure 2 provides no information as to why only a subset of drugs were considered from each country (i.e. 14 of 23 drugs were considered for the US). I expect perhaps that the review period was not available for all drugs, but if that is the case, more information is needed to discuss exclusions, and post-hoc analysis is needed for potential bias due to latent factors related to lack of reporting (i.e. quick withdrawal).

Our response:

Thank you for your important suggestion. Some drugs were excluded from the analysis because data on review periods were not available. For example, 23 drugs received an EUA in the US, of which data on review periods from public websites were available for only 14 drugs. A post-hoc analysis to account for potential bias is out of the scope of our study. In response to your comments, we made the following changes regarding the study’s limitations:

Discussion, Line 330-335

[Before revision]

This study has several limitations. First, it focuses on drugs that were granted the EUA; therefore, it does not consider drugs that were not subject to the EUA but were first approved or granted authorization for use in countries outside the US. Second, the study only addressed pharmaceuticals and did not include medical devices or diagnostic drugs. Third, the countries included in the study were limited to the US and four other countries, excluding other developed or developing nations, which may introduce some variation in global trends.

[After revision]

This study has several limitations. First, it focuses on drugs that were granted the EUA; therefore, it does not consider drugs that were not subject to the EUA, but were first approved or granted authorization for use in countries outside the US. Second, the study only addressed pharmaceuticals and did not include medical devices or diagnostic drugs. Third, the countries included in the study were limited to the US and four other countries, excluding other developed or developing nations, which may introduce some variation in global trends. Fourth, as a result of the limitations mentioned, there may be potential bias in the results.

12. - I expect pairwise t-tests were used to obtain the results between countries. However (1) results are not reported for "non-significant" countries. However, this information must be made available by text or within the figure.

Our response:

Thank you very much for your valuable comments. We conducted the Mann-Whitney U test during the review period and the period from the date of EUA issuance to the date of approval in each country. Consequently, statistically significant differences were found in the review period for US-UK (P=0.0037) and JP-UK (P=0.0391). Since the data on nonsignificant countries were missing, we have added the details of the test results in the text as follows and corrected them:

Results, Lines 205-208

[Before revision]

… The shortest review period was observed in the UK, where the mean was 50.5 days earlier (Fig.2), and a statistically significant difference in the review period was found between the US and the UK (P=0.02).

[After revision]

… The shortest review period was observed in the UK, where the mean was 50.5 days earlier (Fig.2). There was a statistically significant difference in the review period between US-UK (P=0.0037) and JP-UK (P=0.0391). No statistically significant difference in the examination duration was found between US-JP (P=0.9553), US-EU (P=0.4022), JP-EU (P=4.0866), and EU-UK (P=0.1509).

Results, Lines 225-230

[Before revision]

… Although no statistically significant differences were observed, JP had the longest duration from EUA usage permission to approval, with a median of approximately 140 days before approval. As there are only three approved pharmaceuticals in CH, we did not compare its data.

[After revision]

… Although no statistically significant differences were observed, JP had the longest duration from EUA usage permission to approval, with a median of approximately 140 days before approval. As there are only three approved pharmaceuticals in CN, we did not compare this data.

The duration from the date of usage permission granted under the EUA to the approval/usage permission was not a statistically significant difference for all between JP-EU (P=0.9127), JP-UK (P=0.3829), and EU-UK (P=1.7006).

13. - No correction for multiple comparisons was used and must be added to account for the repeated pairwise testing.

Our response:

Thank you for your comment and suggestion. We have accordingly conducted a re-analysis with the Bonferroni correction and tool multiplicity. We made the following corrections:

Statistical methods, Lines 158-162

[Before revision]

Statistical analyses were performed using Microsoft Excel. T-tests were performed for the review periods in each country and the duration from the issuance date of the EUA to the approval date. For the developmental stages of each pharmaceutical in each country, χ2 tests were conducted. Statistical significance was set at 5% (two-tailed).

[After revision]

Microsoft® Excel and R-4.4.1 were used for statistical analysis. The Mann-Whitney U test was used for the review period and the period from the date of EUA issuance to the date of approval in each country, and the Fishers exact test was used for the development stage of each drug in each country. The statistical significance level was set at 5% (two-tailed). Bonferroni correction was applied to the Mann-Whitney U test to account for multiplicity.

Results, Lines 205-208

[Before revision]

… The shortest review period was observed in the UK, where the mean was 50.5 days earlier (Fig.2), and a statistically significant difference in the review period was found between the US and the UK (P=0.02).

[After revision]

… The shortest review period was observed in the UK, where the mean was 50.5 days earlier (Fig.2), There was a statistically significant difference in the review period between US-UK (P=0.0037), and JP-UK (P=0.0391). No statistically significant difference in examination duration was found between US-JP (P=0.9553), US-EU (P=0.4022), JP-EU (P=4.0866), and EU-UK (P=0.1509).

Results, Lines 225-230

[Before revision]

… Although no statistically significant differences were observed, JP had the longest duration from EUA usage permission to approval, with a median of approximately 140 days before approval. As there are only three approved pharmaceuticals in CH, we did not compare its data.

[After revision]

… Although no statistically significant differences were observed, JP had the longest duration from EUA usage permission to approval, with a median of approximately 140 days before approval. As there are only three approved pharmaceuticals in CN, we did not compare its data.

The duration from the date of usage permission granted under the EUA to the approval/usage permission was not a statistically significant difference for all between JP-EU (P=0.9127), JP-UK (P=0.3829), and EU-UK (P=1.7006).

14. - Again, pairwise comparisons data are not provided "no statistically significant differences" is not sufficient without inclusion of data in a table or figure.

Our response:

Thank you for your kind suggestion. We have modified it as follows:

Results, Lines 205-208

[Before revision]

… The shortest review period was observed in the UK, where the mean was 50.5 days earlier (Fig.2), and a statistically significant difference in the review period was found between the US and the UK (P=0.02).

[After revision]

… The shortest review period was observed in the UK, where the mean was 50.5 days earlier (Fig.2). There was a statistically significant difference in the review period between US-UK (P=0.0037) and JP-UK (P=0.0391). No statistically significant difference in examination duration was found between US-JP (P=0.9553), US-EU (P=0.4022), JP-EU (P=4.0866), and EU-UK (P=0.1509).

Results, Lines 225-230

[Before revision]

… Although no statistically significant differences were observed, JP had the longest duration from EUA usage permission to approval, with a median of approximately 140 days before approval. As there are only three approved pharmaceuticals in CH, we did not compare its data.

[After revision]

… Although no statistically significant differences were observed, JP had the longest duration from EUA usage permission to approval, with a median of approximately 140 days before approval. As there are only three approved pharmaceuticals in CN, we did not compare its data.

The duration from the date of usage permission granted under the EUA to the approval/usage permission was not a statistically significant difference for all between JP-EU (P=0.9127), JP-UK (P=0.3829), and EU-UK (P=1.7006).

15. - Were any drugs listed as submitted but not approved during the study period? Given the temporal aspect of this work, and the uncertain timeline of withdrawals and approvals. More consideration may be warranted with survival-type analysis would be appropriate in some cases, for time to event analysis until approval timeframes (rather than simply assuming these models are not ever approved).

- There was also no discussion regarding consideration of temporal analysis. Either in time to event (i.e. approval) survival-type analyses or adjusted regressions accounting for time period and regulatory changes

Our response:

Thank you for your valuable comments. Regarding the temporal analysis, because our main objective was to conduct a US-based comparison with respect to each country's urgent approval system, we tracked key time points, such as approval dates and withdrawal dates, within the study period. For drugs that were submitted to the authorities but not approved during the study period, their status was recorded and considered in the overall analysis. While a detailed analysis with time as a factor, such as a survival analysis, would be beneficial, the main objective of this study was to compare regulatory systems in different countries. Therefore, a descriptive and comparative approach was adopted in this study.

Statistical methods, Lines 158-168

[Before revision]

Statistical analyses were performed using Microsoft Excel. T-tests were performed for the review periods in each country and the duration from the issuance date of the EUA to the approval date. For the developmental stages of each pharmaceutical in each country, χ2 tests were conducted. Statistical significance was set at 5% (two-tailed).

[After revision]

Microsoft® Excel and R-4.4.1 were used for statistical analysis. The Mann-Whitney U test was used for the review period and the period from the date of EUA issuance to the date of approval in each country, and the Fishers exact test was used for the development stage of each drug in each country. The statistical significance level was set at 5% (two-tailed). Bonferroni correction was applied to the Mann-Whitney U test to account for multiplicity.

Regarding the temporal analysis, because our main objective was to make a US-based comparison with respect to each country's urgent approval system, we tracked key time points such as approval dates and withdrawal dates within the study period. For drugs that were submitted to the authorities but not approved during the study period, their status was recorded and considered in the overall analysis. While a detailed analysis with time as a factor, such as a survival analysis, would be beneficial, the main objective of this study was to compare regulatory systems in different countries. Therefore, a descriptive and comparative approach was adopted in this study.

16. The authors touch on many important points. However there remain some disconnections with what was analyzed in this paper. For example, there is no discussion around the differences between "approval vs authorization" and comparisons between countries utilizing each approach.

Our response:

Thank you for your important feedback. In this manuscript, approval refers to the process by which a drug meets full regulatory requirements and is authorized for permanent use. Use authorization is the process by which a drug is allowed to be used temporarily when a rapid response is required, such as in an emergency, and is usually based on limited data. Due to a lack of explanation, the following corrections have been made:

Results, Lines 241-250

[Before revision]

An overview of the investigation of emergency approval/usage permission systems in each country is presented in Table 3. Approved drugs (EU: Conditional Marketing Authorisations, JP: Special Approval for Emergency, CH: Conditional Market Approval) can be used even after an emergency situation has passed. However, use authorization (US: Emergency Use Authorization, UK: Temporary Use Authorisation, CH: Chinese Vaccine Management Law) is terminated after an emergency situation has passed, and the drug can no longer be used. The most significant difference in each country’s system was whether it operated under an approval or a usage permission framework....

[After revision]

　An overview of the investigation of emergency approval/usage permission systems in each country is presented in Table 3. Drugs approved with expedited programs (EU: Conditional Marketing Authorisations, JP: Special Approval for Emergency, CN: Conditional Market Approval) can be used even after an emergency situation has passed. However, use authorization (US: Emergency Use Authorization, UK: Temporary Use Authorisation, CH: Chinese Vaccine Management Law) is terminated after an emergency situation has passed, and the drug can no longer be used. The most significant difference in each country’s system was whether it operated under an approval or a usage permission framework. Approval refers to the process by which a drug meets full regulatory requirements and is authorized for permanent use. Use authorization is the process by which a drug is allowed to be used temporarily when a rapid response is required, such as in an emergency, and is usually based on limited data. The added data may trigger a move to ordinary full approval or withdrawal of permission to use.

Results, Lines 246-251

[Before revision]

…The most significant difference in each country’s system was whether it operated under an approval or a usage permission framework. The US and UK employed a usage permission system, …

[After revision]

…The most significant difference in each country’s system was whether it operated under an approval or a usage permission framework. Approval refers to the process by which a drug meets full regulatory requirements and is authorized for permanent use. Use authorization is the process by which a drug is allowed to be used temporarily when a rapid response is required, such as in an emergency, and is usually based on limited data. The added data may trigger a move to ordinary full approval or withdrawal of permission to use. The US and UK employed a usage permission system …

…

17. - The authors also note the addition of specific amendments that may have impacted timelines, as such I would have expected adjusted analysis to account for such.

Our response:

Thank you for your valuable comments. Regarding the temporal analysis, because our main objective was to conduct a US-based comparison with respect to each country's urgent approval system, we tracked key time points such as approval dates and withdrawal dates within the study period. For drugs that were submitted to the authorities but not approved during the study period, their status was recorded and considered in the overall analysis. While a detailed analysis with time as a factor, such as a survival analysis, would be beneficial, the main objective of this study was to compare regulatory systems in different countries. Therefore, a descriptive and comparative approach was adopted in this study.

Statistical methods Lines 158-168

[Before revision]

Statistical analyses were performed using Microsoft Excel. T-tests were performed for the review periods in each country and the duration from the issuance date of the EUA to the approval date. For the developmental stages of each pharmaceutical in each country, χ2 tests were conducted. Statistical significance was set at 5% (two-tailed).

[After revision]

Microsoft® Excel and R-4.4.1 were used for statistical analysis. The Mann-Whitney U test was used for the review period and the period from the date of EUA issuance to the date of approval in each country, and the Fishers exact test was used for the development stage of each drug in each country. The statistical significance level was set at 5% (two-tailed). Bonferroni correction was applied to the Mann-Whitney U test to account for multiplicity.

Regarding the temporal analysis, because our main objective was to make a US-based comparison with respect to each country's urgent approval system, we tracked key time points such as approval dates and withdrawal dates within the study period. For drugs that were submitted to the authorities but not approved during the study period, their status was recorded and considered in the overall analysis. While a detailed analysis with time as a factor, such as a survival analysis, would be beneficial, the main objective of this study was to compare regulatory systems in different countries. Therefore, a descriptive and comparative approach was adopted in this study.

III. Response to Reviewer #2

1. In the Discussion, they stated that an emergency approval/use authorization system must be effective in every country. I am not sure what does it mean by “effective” in their terms, and I assumed “effective” means “having a good effect.” It could be very interesting if they specified the characteristics of “effective regulatory frameworks” in emergency situations. This is just a suggestion, but it would be appreciated if they gave some opinions about this point.

Our response:

Thank you for your valuable suggestion. We believe that "effective regulatory frameworks" means regulatory frameworks characterized by "expedited approval/use permission processes," "balance between safety and efficacy," "flexible data requirements," "transparency," and

"pharmacovigilance."

Discussion, Lines 313-329

[Before revision]

We believe that such a system must be effective in every country.

[After revision]

We believe that such a system must be effective in every country. In emergency situations, an expedited approval process is required. This includes special procedures that simplify and expedite the ordinary approval process, for example, the EUA in the U.S. and the CMA in the EU. Such schemes are essential to enable rapid access to medicines in emergency situations and to respond to crises such as pandemics. It is also important to strike a balance between safety and efficacy. The emergency approval process must ensure that known and potential risks are minimal, even when approvals are based on limited data [25]. This ensures that treatments are safe and effective for patients, even in emergency situations. Additionally, flexible data requirements are necessary. In emergency situations, approvals must be based on limited data without waiting for complete clinical data [25]. This includes flexible data requirements that simplify the documentation required for applications and allow for expedited review. Additionally, transparency is critical. The emergency approval process requires that authorities set clear criteria for the approval process and decisions and provide appropriate information to healthcare professionals and the public [26]. This ensures that reliable information is shared, and effective treatment is provided during emergencies. Finally, pharmacovigilance of emergency-approved drugs is also important. Pharmacovigilance is executed and, if necessary, the approval is reviewed or withdrawn to continuously assess safety and efficacy.

A regulatory framework with the above features will function effectively in emergency situations, enabling the prompt and safe delivery of medicines.

2. Please use CN for the abbreviation of China.

Our response:

Thank you for pointing this out. We have corrected the abbreviation CN for China in the entire paper. We have also revised Table 1, Table 2, and Table 3 to use CN as the abbreviation for China. The corrected parts are shown below.

Abstract, Lines 39, 41, 43, 44, 48-49

[Before revision]

…United Kingdom (UK), and China (CH) for pharmaceuticals (n=23) …

…JP had granted approval or permission for 14 drugs (60.9%), EU for 14 (60.9%), UK for 12 (52.2%), and CH for…

…the EU had 3 drugs (8.7%), the UK had 5 drugs (21.7%), and CH had 16 drugs (69.6%) yet to be developed. …

…US had significantly more granted permissions and developed drugs, while CH the least. …

…UK were developed or on the market in over 70% of cases, whereas in CH, more than two-thirds were yet to be developed. This suggests that CH may not actively utilize pharmaceuticals from other countries for COVID-19…

[After revision]

…United Kingdom (UK), and China (CN) for pharmaceuticals (n=23) …

…JP had granted approval or permission for 14 drugs (60.9%), EU for 14 (60.9%), UK for 12 (52.2%), and CN for …

…the EU had 3 drugs (8.7%), the UK had 5 drugs (21.7%), and CN had 16 drugs (69.6%) yet to be developed. …

…US had significantly more granted permissions and developed drugs, while CN the least. …

…UK were developed or on the market in over 70% of cases, whereas in CN, more than two-thirds were yet to be developed. This suggests that CN may not actively utilize pharmaceuticals from other countries for COVID-19…

Introduction, Lines 81, 84, 95

[Before revision]

… Kingdom (UK) [7], and China (CH) [8], early approval and permission systems have been established as …

… in JP, the US, the EU, the UK, and CH, focusing on investigating the approval and …

… approved or permitted in other countries (JP, EU, UK, and CH) and conducts a comparative analysis of …

[After revision]

… Kingdom (UK) [7], and China (CN) [8], early approval and permission systems have been established as …

… in JP, the US, the EU, the UK, and CN, focusing on investigating the approval and …

… approved or permitted in other countries (JP, EU, UK, and CN) and conducts a comparative analysis of …

Materials and methods, Lines 103

[Before revision]

… Information on pharmaceuticals granted usage permission under the US Emergency Use Authorization (EUA) and situations in other countries (the US, JP, EU, UK, and CH) …

[After revision]

… We identified emergency approval/usage permission systems in each country (the US, JP, EU, UK, and CN) …

Results, Lines 188, 192-193

[Before revision]

… for 14 drugs (60.9%), the EU for 14 drugs (60.9%), the UK for 12 drugs (52.2%), and CH for 3 drugs (13.0%). …

… the UK had five drugs (21.7%), JP had six drugs (26.1%), and CH had 16 drugs (69.6%) that were yet to be developed. The proportion of undeveloped pharmaceuticals in CH was significantly larger compared to other …

[After revision]

… for 14 drugs (60.9%), the EU for 14 drugs (60.9%), the UK for 12 drugs (52.2%), and CN for 3 drugs (13.0%). …

… the UK had five drugs (21.7%), JP had six drugs (26.1%), and CN had 16 drugs (69.6%) that were yet to be developed. The proportion of undeveloped pharmaceuticals in CN was significantly larger compared to other …

Results, Lines 208

[Before revision]

… US-JP (P=0.9553), US-EU (P=0.4022), JP-EU (P=4.0866), and EU-UK (P=0.1509). As for the data on CH, …

[After revision]

… US-JP (P=0.9553), US-EU (P=0.4022), JP-EU (P=4.0866), and EU-UK (P=0.1509). As for the data on CN, …

Results, Lines 227

[Before revision]

… As there are only three approved pharmaceuticals in CH, we did not compare this data.

[After revision]

… As there are only three approved pharmaceuticals in CN, we did not compare this data.

Results, Lines 243-244, 251-252, 260-262

[Before revision]

… Approval for Emergency, CH: Conditional Market Approval) can be used even after an emergency situation has passed. However, use authorization (US: Emergency Use Authorization, UK: Temporary Use Authorisation, CH: Chinese Vaccine Management Law). …

… and UK employed a usage permission system, whereas JP, the EU, and CH operated under an approval system. Additionally, in JP, the UK, and CH, new systems were established in response to the COVID-19 pandemic …

... The CN introduced a Vaccine Administration Law enabling the emergency use of vaccines and implemented conditional market approval for CH in 2019 [8].

[After revision]

… Approval for Emergency, CN: Conditional Market Approval) can be used even after an emergency situation has passed. However, use authorization (US: Emergency Use Authorization, UK: Temporary Use Authorisation, CN: Chinese Vaccine Management Law). …

… and UK employed a usage permission system, whereas JP, the EU, and CN operated under an approval system. Additionally, in JP, the UK, and CN, new systems were established in response to the COVID-19 pandemic …

... The CN introduced a Vaccine Administration Law enabling the emergency use of vaccines and implemented conditional market approval for CN in 2019 [8].

Discussion, Lines 290

[Before revision]

…Administration (NMPA) implemented conditional market approval for CH in 2019 [8].

[After revision]

…Administration (NMPA) implemented conditional market approval for CN in 2019 [8].

Discussion, Lines 297-302

[Before revision]

… EUA for use from December 1, 2019, to July 31, 2023, in JP, the EU, the UK, and CH. As a result, more than 70% of the pharmaceuticals authorized by the EUA for COVID-19 had developed or were in the market in JP, the EU, and the UK. In contrast, CH has over two-thirds of its pharmaceuticals yet to be developed, suggesting a distinct approach from other countries. Therefore, CH may have independently developed its own pharmaceuticals for the COVID-19 pandemic. Furthermore, in terms of pharmaceuticals for COVID-19, 19 of 23 pharmaceuticals authorized by the EUA had received authorization in the US, surpassing JP, the EU, the UK, and CH.

[After revision]

… EUA for use from December 1, 2019, to July 31, 2023, in JP, the EU, the UK, and CN. As a result, more than 70% of the pharmaceuticals authorized by the EUA for COVID-19 had developed or were in the market in JP, the EU, and the UK. In contrast, CN has over two-thirds of its pharmaceuticals yet to be developed, suggesting a distinct approach from other countries. Therefore, CN may have independently developed its own pharmaceuticals for the COVID-19 pandemic. Furthermore, in terms of pharmaceuticals for COVID-19, 19 of 23 pharmaceuticals authorized by the EUA had received authorization in the US, surpassing JP, the EU, the UK, and CN

Conclusions, Lines 339-342

[Before revision]

… pharmaceuticals during emergencies in Japan, the United States, Europe, and China (JP, US, EU, UK, and CH), focusing on 23 pharmaceuticals authorized for use against COVID-19 through the EUA granted by the FDA. While over 70% of the EUA-approved drugs have been developed or marketed in the JP, EU, and UK, more than 2/3 of them are yet to be developed for CH. This suggests that CH may not …

[After revision]

… pharmaceuticals during emergencies in Japan, the United States, Europe, and China (JP, US, EU, UK, and CN), focusing on 23 pharmaceuticals authorized for use against COVID-19 through the EUA granted by the FDA. While over 70% of the EUA-approved drugs have been developed or marketed in the JP, EU, and UK, more than 2/3 of them are yet to be developed for CN. This suggests that CN may not …

3. Table 1: “Total” column is confusing, so the column can be omitted. It appears that the Total column is also included in the Chi-squared test.

Our response:

Thank you for your important feedback. We have removed the "Total" column from Table 1. Also, the statistical test was conducted without including the "Total" column.

4. Table 1: Row name “Withdrawal” is not appropriate, so please consider renaming . I assumed that that category contains drugs that are still yet to be developed

Our response:

Thank you for your attention to this important point. As you pointed out, the item "withdrawal" in Table 1 includes drugs that have not yet been developed, and we have revised the item "withdrawal" in Table 1 to "withdrawal or undeveloped" and modified Table 1 as shown in the attached file.

5. Table 2: Notation of p value is not clear. P< .0001 (the first 0 is missing). In Table 1, there is 0 in front of the decimal point. Please unify the notation.

Our response:

Thank you for your valuable comments. We have corrected the lack of consistency in the notation of P values in Table 1 and Table 2.

6. They stated in Line 154: “The proportion of undeveloped pharmaceuticals in CH was significantly larger compared to other countries (P<0.0001) (Table 2).” It appears that the P value supports the large proportion of the undeveloped pharmaceuticals, which is not the case, I believe. Chi-squared test on Table 2 is merely the difference of the percentage is statistically significant along with countries or development status. Chi-squared test does not imply anything on the specific columns or rows. Please state the precise implication of Chi-squared test with p-value, and then state about specific column and/or row.

Our response:

Thank you for your valuable input. As you pointed out, the chi-square test does not indicate anything about a particular column or row. The P value of the Chi-square test (P<0.0001) indicates that the proportion of undeveloped drugs in each country is not uniform. The statistical method was changed, and the Fishers exact test was performed for the development stage of each drug in each country, which was corrected as follows:

Results, Lines 189– 196

[Before revision]

… Additionally, an investigation into the developmental status of pharmaceuticals granted usage permission under the EUA as of the end of July 2023 in each country showed that the EU had three drugs (8.7%), the UK had five drugs (21.7%), JP had six drugs (26.1%), and CH had 16 drugs (69.6%) that were yet to be developed. The proportion of undeveloped pharmaceuticals in CH was significantly larger compared to other countries (P<0.0001) (Table 2). …

[After revision]

… Additionally, an investigation into the developmental status of pharmaceuticals granted usage permission under the EUA as of the end of July 2023 in each country showed that the EU had three drugs (8.7%), the UK had five drugs (21.7%), JP had six drugs (26.1%), and CN had 16 drugs (69.6%) that were yet to be developed. The results of the Fishers exact test showed a statistically significant difference (P<0.0001) in "Development status in each country for drugs granted the Emergency Use Authorization by the FDA" (Table 2). This indicates that the development status of drugs is not uniform across countries. In particular, CN has a significantly higher percentage of undeveloped drugs than other countries, suggesting that there may be differences in drug development processes and priorities.

7. In Figure 2, authors compared the review periods in four countries and conducted even statistical test between the US and UK. I believe the comparison is not meaningful because the US already gave permission to use it with the EUA. The situation is somewhat different. My suggestion is to compare three countries JP, EU, and UK, instead.

Our response:

Thank you for your valuable suggestions. The "review periods" mean the period of timing between the receipt date of the application for approval/use permit and the granted date of the first approval/use permit. For example, for the US, it refers to the period from the EUA Receipt Date to the date the use permit was granted. The "review periods" mean the period of timing between the receipt date of the application for approval/use permit and the granted date of the first approval/use permit. For example, for the US, it refers to the period from the EUA receipt date to the date the use permit was granted.

Data Collection, Lines 104 – 133

[Before revision]

… The specific investigation parameters included approval or usage permission status, approval or usage permission dates, JP approval application dates, expiration dates of approval or usage permission, review periods, the duration from EUA issuance to approval or usage permission date in each country, and the developmental stage. Data collection was primarily conducted through the official websites of regulatory authorities in each country [11–15]. Each website was reviewed, and the data were manually extracted and verified by a researcher.

[After revision]

… The specific investigation parameters included approval or usage permission status, approval or usage permission dates, JP approval application dates, expiration dates of approval or usage permission, review periods, the duration from EUA issuance to approval or usage permission date in each country, and the developmental stage. Data collection was primarily conducted through the official websites of regulatory authorities in each country [11–15]. Each website was reviewed, and the data were manually extracted and verified by a researcher. Data on the status of approval/use authorization, date of approval/use authorization, date of JP approval, date of withdrawal of approval/use authorization, review period, and period from the date of EUA issuance to the date of approval/use authorization in each country were collected from the regulatory agency web sites in each country as shown below. We also collected data from ClinicalTrials.gov on the pre-market phase of the drug, the most recent data available as of July 31, 2023. Duplicates were eliminated in the collected data, formatted uniformly, and compiled into a database.

In the United States (US): Information was collected from Drugs@FDA, the Emergency Use Authorization (EUA) page of the FDA web site, and FDA press releases.

(<https://www.fda.gov/emergency-preparedness-and-response/mcm-legal-regulatory-and-policy-framework/emergency-use-authorization>)

In Japan (JP), information was collected from the Pharmaceuticals and Medical Drugs Directory (PMDA), the Ministry of Health, Labour and Welfare (MHLW) website, and MHLW press releases.

(<https://www.pmda.go.jp/PmdaSearch/iyakuSearch>, <https://www.mhlw.go.jp/index.html>)

Europe (EU): Information was collected from the Conditional Marketing Authorization (CMA) page of the EMA web site and EMA press release information.

(<https://www.ema.europa.eu/en/glossary-terms/conditional-marketing-authorisation>)

In United Kingdom (UK): Information was collected from MHRA Products Substance Index, MHRA web site page on Temporary Use authorization, and MHRA press release information.

(<https://www.gov.uk/government/publications/freedom-of-information-responses-from-the-mhra-week-commencing-14-march-2022/freedom-of-information-request-on-the-different-legal-classifications-for-vaccines-foi-21415>)

CHINA (CN): Information was collected from the official NMPA website, CENTER FOR DRUG EVALUATION, NMPA.

(<https://english.nmpa.gov.cn/index.html>, <https://english.nmpa.gov.cn/2019-07/19/c_389169.htm>)

8. Line 172 and Line 190: The x-axis represents the average values. -> The x mark represents…

Our response:

Thank you for your comments. We have made the following corrections:

Results, Lines 214

[Before revision]

… The x-axis represents the average values.

[After revision]

… The x mark represents the average values.

Results, Lines 234

[Before revision]

… The x-axis represents the average values.

[After revision]

… The x mark represents the average values.

9. Line 197: Approved drugs (EU: Conditional Marketing Authorisations, JP: Special Approval for Emergency, CH: Conditional Market Approval) can be used… Each item inside of the parenthesis should be corresponding to the word immediately before, “drugs”. Please consider re-phrase like this:

-> Drugs approved with expedited programs (EU: Conditional Marketing Authorisations, JP: Special Approval for Emergency, CH: Conditional Market Approval) can be used even after …

Our response:

Thank you for sharing your consideration. We have made the following corrections:

Results, Lines 241-244

[Before revision]

…in Table 3. Approved drugs (EU: Conditional Marketing Authorisations, JP: Special Approval for Emergency, CH: Conditional Market Approval) can be used even after an emergency situation has passed. However, …

[After revision]

…in Table 3. Drugs approved with expedited programs (EU: Conditional Marketing Authorisations, JP: Special Approval for Emergency, CN: Conditional Market Approval) can be used even after an emergency situation has passed. However, …

10. Paragraph: 4. System for approval/usage permission under …

What is the difference among approval, use authorization, and usage permission. I could not follow the discussion.

Our response:

Thank you for your important feedback. In this manuscript, approval refers to the process by which a drug meets full regulatory requirements and is authorized for permanent use. Use authorization is the process by which a drug is allowed to be used temporarily when a rapid response is required, such as in an emergency, and is usually based on limited data. Due to a lack of explanation, the following has been corrected:

Results, Lines 241-250

[Before revision]

An overview of the investigation of emergency approval/usage permission systems in each country is presented in Table 3. Approved drugs (EU: Conditional Marketing Authorisations, JP: Special Approval for Emergency, CH: Conditional Market Approval) can be used even after an emergency situation has passed. However, use authorization (US: Emergency Use Authorization, UK: Temporary Use Authorisation, CH: Chinese Vaccine Management Law) is terminated after an emergency situation has passed, and the drug can no longer be used. The most significant difference in each country’s system was whether it operated under an approval or a usage permission framework.…

[After revision]

　An overview of the investigation of emergency approval/usage permission systems in each country is presented in Table 3. Drugs approved with expedited programs (EU: Conditional Marketing Authorisations, JP: Special Approval for Emergency, CH: Conditional Market Approval) can be used even after an emergency situation has passed. However, use authorization (US: Emergency Use Authorization, UK: Temporary Use Authorisation, CN: Chinese Vaccine Management Law) is terminated after an emergency situation has passed, and the drug can no longer be used. The most significant difference in each country’s system was whether it operated under an approval or a usage permission framework. Approval refers to the process by which a drug meets full regulatory requirements and is authorized for permanent use. Use authorization is the process by which a drug is allowed to be used temporarily when a rapid response is required, such as in an emergency, and is usually based on limited data. The added data may trigger a move to ordinary full approval or withdrawal of permission to use.

Results, Lines 246-251

[Before revision]

…The most significant difference in each country’s system was whether it operated under an approval or a usage permission framework. The US and UK employed a usage permission system, …

[After revision]

…The most significant difference in each country’s system was whether it operated under an approval or a usage permission framework. Approval refers to the process by which a drug meets full regulatory requirements and is authorized for permanent use. Use authorization is the process by which a drug is allowed to be used temporarily when a rapid response is required, such as in an emergency, and is usually based on limited data. The added data may trigger a move to ordinary full approval or withdrawal of permission to use. The US and UK employed a usage permission system, …

…

11. The first paragraph of the Discussion can be included in the Results section, explaining about Table 3.

Our response:

Thank you for your kind comments. We have extracted a section from the first paragraph of the Discussion and added it to the Results section. The following corrections have been made:

Results, Lines 252-263

[Before revision]

… In JP, an emergency approval system has been introduced, allowing the approval of pharmaceuticals not approved overseas. In the UK, a conditional marketing authorization system was implemented. The CH introduced a Vaccine Administration Law enabling the emergency use of vaccines.

[After revision]

… In JP, an emergency approval system has been introduced, allowing the approval of pharmaceuticals not approved overseas. In JP, a 'Special Approval System' initially existed as an emergency approval system for drugs. This system aims to expedite the regular review process and approve products for market use in cases of urgency or medical necessity. As the Special Approval System primarily focuses on approving overseas drugs, a new emergency approval system capable of approving JP-origin pharmaceuticals was established in 2023 [10]. In the UK, a conditional marketing authorization system was implemented. Additionally, the Medicines and Healthcare Products Regulatory Agency (MHRA) established Temporary Use Authorization in 2020. This authorization simplifies the regular review process, enabling rapid commercialization through use authorization [7]. The CN introduced a Vaccine Administration Law enabling the emergency use of vaccines and implemented conditional market approval for CN in 2019 [8]. This system expedites the progress of the regular review process to meet urgent medical needs, allowing early patient access to pharmaceuticals.

End of text
